# Impact of number of functional teeth on independence of Japanese older adults

**DOI:** 10.1111/ggi.14508

**Published:** 2022-11-21

**Authors:** Kenji Maekawa, Tomoko Ikeuchi, Shoji Shinkai, Hirohiko Hirano, Masahiro Ryu, Katsushi Tamaki, Hirofumi Yatani, Takuo Kuboki, Kusatsu ISLE Study Working Group Collaborators, Aya Kimura‐Ono, Takeshi Kikutani, Takashi Suganuma, Yasunori Ayukawa, Tomoya Gonda, Toru Ogawa, Masanori Fujisawa, Shoichi Ishigaki, Yutaka Watanabe, Akihiko Kitamura, Yu Taniguchi, Yoshinori Fujiwara, Ayako Edahiro, Yuki Ohara, Junichi Furuya, Junko Nakajima, Kento Umeki, Kentaro Igarashi, Yasuhiro Horibe, Yoshihiro Kugimiya, Yasuhiko Kawai, Hideo Matsumura, Tetsuo Ichikawa, Shuji Ohkawa, Kazuyoshi Baba

**Affiliations:** ^1^ Research Planning and Promotion Committee Japan Prosthodontic Society Tokyo Japan; ^2^ Okayama University Graduate School of Medicine, Dentistry, and Pharmaceutical Sciences Okayama Japan; ^3^ Tokyo Metropolitan Institute of Gerontology Tokyo Japan; ^4^ Kagawa Nutrition University Sakado Japan; ^5^ Tokyo Dental College Tokyo Japan; ^6^ Kanagawa Dental University Graduate School Yokosuka Japan; ^7^ Osaka University Graduate School of Dentistry Osaka Japan; ^8^ Okayama University Hospital Okayama Japan; ^9^ The Nippon Dental University Tokyo Japan; ^10^ Showa University School of Dentistry Tokyo Japan; ^11^ Kyushu University Faculty of Dental Science Fukuoka Japan; ^12^ Tohoku University Graduate School of Dentistry Sendai Japan; ^13^ Meikai University School of Dentistry Sakado Japan; ^14^ Faculty of Dental Medicine Hokkaido University Sapporo Japan; ^15^ National Institute for Environmental Studies Ibaraki Japan; ^16^ Nihon University School of Dentistry at Matsudo Chiba Japan; ^17^ Nihon University School of Dentistry Tokyo Japan; ^18^ Tokushima University Graduate School Institute of Biomedical Sciences Tokushima Japan

**Keywords:** community‐dwelling older adults, functional teeth, loss of independence, oral health, present teeth

## Abstract

**Aim:**

To examine the relationship between the number of present and functional teeth at baseline and future incidence of loss of independence.

**Methods:**

Participants were community‐dwelling older individuals who participated in a comprehensive geriatric health examination conducted in Kusatsu town, Japan, between 2009 and 2015. The primary endpoint was the incidence of loss of independence among participants, defined as the first certification of long‐term care insurance in Japan. The numbers of present and functional teeth at baseline were determined via an oral examination. Demographics, clinical variables (e.g., history of chronic diseases and psychosocial factors), blood nutritional markers, physical functions, and perceived masticatory function were assessed.

**Results:**

This study included 1121 individuals, and 205 individuals suffered from loss of independence during the follow‐up period. Kaplan–Meier estimates of loss of independence for participants with smaller numbers of present and functional teeth were significantly greater than for those with larger numbers of teeth. Cox proportional hazard analyses indicated that a smaller number of present teeth was not a significant risk factor after adjusting for demographic characteristics. However, the number of functional teeth was a significant risk factor after the adjustment (hazard ratio: 1.975 [1.168–3.340]). Additionally, higher hazard ratios were observed in other adjusted models, but they were not statistically significant.

**Conclusions:**

The number of functional teeth may be more closely related to the future incidence of loss of independence than the number of present teeth. This novel finding suggests that prosthodontic rehabilitation for tooth loss possibly prevents the future incidence of this life‐event. **Geriatr Gerontol Int 2022; 22: 1032–1039**.

Loss of independence (LOI) is an important life‐event, leading to the loss of healthy life expectancy and approaching mortality, and it should be prevented. In this situation, oral function plays an important role in the intake of nutrients necessary for health maintenance and therefore is associated with the physical and cognitive functioning of older adults. Significant associations have been observed between frailty and reduction in the number of present teeth (NPT), occlusal force, mixing ability, self‐perceived chewing ability, oral diadochokinesis score, and masseter muscle thickness.^1^, ^2^, ^3^ Moreover, recent prospective cohort studies have demonstrated significant associations of the incidence of LOI in older individuals with a smaller NPT^4^ and the reduction of occlusal support.^5^ Previous studies presumed that a reduction in the NPT induces nutritional and physical decline, which leads to LOI.

However, most people with loss of teeth receive prosthetic rehabilitation to increase the number of functional teeth (NFT). Thus, LOI can be prevented with prosthodontic treatment and the maintenance of the nutritional status. Indeed, our recent study revealed that the NFT more strongly predicts all‐cause mortality than the NPT among community‐dwelling older adults.^6^ This suggests that increasing the NFT through prosthetic rehabilitation could mitigate or cancel the negative effects of fewer present teeth (PT) on the risk of mortality by improving masticatory function and nutritional status. Furthermore, increasing the NFT would have a positive effect on the prevention of LOI, but no studies have examined this association.

In this study, we investigated the relationship between the NFT and the incidence of LOI in community‐dwelling older adults. In particular, we examined the relationship of the NPT and NFT at baseline with the future incidence of LOI, adjusted for multiple factors, adjusting for multiple factors including demographics, history of chronic disease, nutritional, psychosocial and physical status, and subjectively evaluated masticatory function.

## Materials and methods

### Participants

Most participants from those who participated in a comprehensive geriatric health examination for community‐dwelling older individuals (≥65 years) conducted in Kusatsu town, Gunma, Japan, between 2009 and 2015. Most participants completed this examination multiple times, with the examination being held annually in the summer during the sampling period at the same local public health center. Therefore, data from the first examination of each participant during this period were regarded as the baseline data. We enrolled 1240 individuals (539 men and 701 women; mean age, 76.6 ± 7.1 years), who were informed of the purpose, nature, and potential risks of the examination prior to providing consent for the study. The study protocol was reviewed and approved by the Ethics Committee of the Tokyo Metropolitan Institute of Gerontology, in accordance with the Declaration of Helsinki.

### Endpoint and follow up

The study endpoint was the incidence of LOI among the participants, which was defined as a new certification of long‐term care insurance (LTCI)^7^ or death before receiving LTCI certification.^8^ LTCI is a universal system across Japan, and in each municipality the Local Certification Committee determines who should receive LTCI from applicants experiencing difficulties in their activities of daily living. Mortality was confirmed by checking local registries linking with the Japanese National Vital Statistics.

The follow‐up period was up to February 2016. Participants who could not continue follow‐up owing to relocation were excluded from the study. Those who had already experienced LOI at the time of the baseline examination were also excluded.

### Examination items

Data regarding the following variables were recorded: age, sex, history of chronic diseases, Geriatric Depression Scale (GDS) short version score,^9^ Mini‐Mental State Examination (MMSE) score,^10^ body mass index (BMI), blood nutritional markers, physical function (gait speed and grip strength), and self‐perceived chewing ability.^11^ The presence of the following clinically relevant chronic diseases was examined: hypertension, hyperlipidemia, cerebral vascular disease, heart disease, diabetes mellitus, and cancer. All participants were asked if they had received a clinical diagnosis for each pathological condition. GDS was evaluated using a self‐administered questionnaire consisting of 15 items with “yes” or “no” answers, and each negative answer was assigned a score of 1. The total score indicated the participants' depression level; the maximum score was 15. A GDS score cut‐off value of 6 was used to separate participants into two groups: <6 and ≥6.^9^ The MMSE is widely used to evaluate cognitive function and as a screening test for dementia;^10^ the score of each participant was determined during a face‐to‐face interview. The MMSE consists of 11 questions with a 30‐point scale; the total score represents a participant's overall cognitive function, and a cut‐off value of 24 was used to separate participants into two groups: <24 and ≥24.^12^ Height and weight were measured, and peripheral blood was collected for testing. Participants were categorized into three groups according to BMI (<20.0, 20.0–24.9, and ≥25.0 kg/m^2^).^13^ Regarding blood nutritional markers, participants were categorized into two groups (hemoglobin, male [<13.0 and ≥13.0 g/dL] and female [<12.0 and ≥12.0 g/dL];^14^ albumin [<4.0 and ≥4.0 g/dL];^15^ and total cholesterol, male [<160 and ≥160 mg/dL] and female [<180 and ≥180 mg/dL]). Lower cholesterol levels in older adults are associated with an increased risk of mortality.^16^ Therefore, we applied <160 and <180 mg/dL as the cut‐off values for total cholesterol in men and women, respectively, based on data from the National Health and Nutrition Survey Japan.

To evaluate each participant's physical function, gait speed and grip strength were measured. Usual gait speed was measured according to the method described by Nofuji *et al*.,^17^ and participants were divided into two categories: <1.0 and ≥1.0 m/s.^18^ The grip strength of each participant's dominant hand was measured using a Smedley‐type hand dynamometer (Yamagami Co, Tokyo, Japan). The higher value from two trials was considered to represent the participant's grip strength. Participants were categorized into two groups: men, >26.0 and ≤26.0 kgf; women, >18.0 and ≤18.0 kgf.^19^ Self‐perceived chewing ability was assessed with the following question:^11^ “Do you experience difficulties in chewing food? If you use dentures, please answer this question assuming that dentures are used.” Participants selected one answer from among four choices: (i) no difficulties; (ii) some difficulties, but can eat most food; (iii) difficulties, and can eat only limited food; and (iv) cannot chew much. Because only three participants chose (iv), those who chose (iii) or (iv) were grouped together for subsequent analyses.

### Oral examination

Oral examination was performed by experienced dentists. To standardize each examiner's competence, a calibration procedure was performed before the examination. The NPT and NFT were counted for each participant. The detailed counting methods of NPT and NFT are described by Maekawa *et al*.^6^ Based on the NPT, we categorized participants into three groups:^6^, ^20^ 0–9, 10–19, and ≥20 teeth. Several studies have suggested that the presence of ≥20 teeth enables satisfactory chewing ability.^21^ Participants were also categorized into two groups based on the NFT: ≤19 and ≥20 teeth.^6^

### Statistical analysis

Chi‐square tests and t‐tests were used to compare baseline predictors between the two groups (categorized by the NFT). Kaplan–Meier analysis was used to estimate the incidence of LOI. The log‐rank test was used to compare the LOI curves between or among groups. Finally, Cox proportional hazard models were used to calculate the hazard ratio (HR) for LOI. Six analytical models were used in this study. This modeling strategy was based on the stepwise increase of predictors as potential risk factors for decline in activities of daily living. They were carried out with the adjustment of demographic characteristics. Because our previous study suggested that self‐chewing ability at baseline was associated with the future incidence of functional decline,^11^ this factor was assumed to correlate with the incidence of LOI. Thus, it was submitted to the last statistical model, Model 6. Model 0 (crude, unadjusted model): only the NPT and NFT were simultaneously submitted as predictors; Model 1: Model 0, adjusted for demographic characteristics (age and sex); Model 2: Model 1, adjusted for clinically relevant variables (hypertension, hyperlipidemia, stroke, heart disease, diabetes, cancer, depression, and cognitive function); Model 3: Model 2, adjusted for BMI and nutritional markers (albumin, hemoglobin, and cholesterol); Model 4: Model 3, adjusted for physical function (usual gait speed and hand grip strength); and Model 5: Model 4, adjusted for self‐perceived chewing ability. The significance level was set at *P* < 0.05. All statistical analyses were performed using IBM SPSS Statistics (version 25.0., IBM Corp., Armonk, NY).

## Results

### Study flow

**Figure 1:**
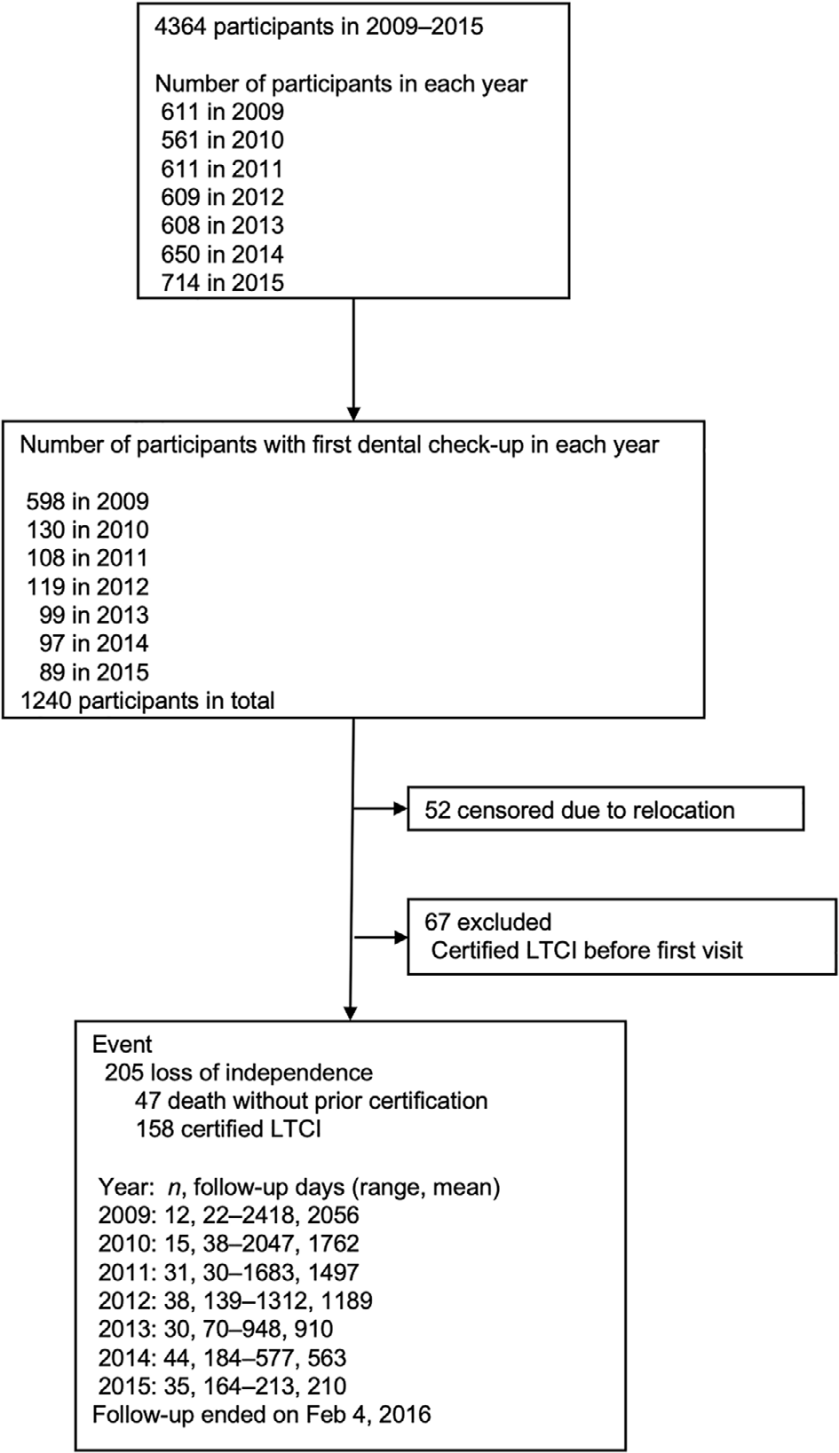
Flow diagram for the selection of study participants.

In total, 4364 individuals participated in the annual health examinations during the study period. We enrolled 1240 individuals who completed the first health examination including an oral examination during the period. Participants who could not complete follow‐up owing to relocation (*n* = 52) or who had already received LTCI certification at baseline (*n* = 67) were excluded from this study. Therefore, 1121 individuals were finally included in the analysis (Fig. 1).

### Sample characteristics

Table 1 shows the baseline characteristics of the participants according to the NFT (0–19 and ≥20). Significant baseline differences were observed between the NFT and other factors, such as the NPT distribution, sex, a history of hypertension and hyperlipidemia, blood nutritional parameters (hemoglobin), and self‐perceived chewing ability. These data suggest that the NFT independently influenced some of the potential risk factors for the incidence of LOI. The baseline data of the participant groups categorized by the future incidence of LOI is provided in the Supplementary Material (Table S1).

**Table 1:** Comparisons of baseline properties between the groups categorized by the number of functional teeth

Variables	Number of functional teeth				
	0–19 (*n* = 58)		≥20 (*n* = 1063)		*P*‐value
Loss of independence (LOI), *n* (%)	16	(27.6)	189	(17.8)	0.060^†^
Incidence rate of LOI per 1000 person‐years	71		41		
Number of present teeth					**<0.001** ^†^
0–9, *n* (%)	31	(53.4)	395	(37.2)	
10–19, *n* (%)	27	(46.6)	209	(19.7)	
≥20, *n* (%)	0	0	458	(43.1)	
Age (years), mean (SD)	73	(6.5)	71.7	(6.6)	0.840^‡^
Sex (male), *n* (%)	33	(56.9)	458	(43.1)	**0.039** ^†^
History of chronic disease					
Hypertension, *n* (%)	15	(25.9)	429	(40.4)	**0.027** ^†^
Hyperlipidemia, *n* (%)	5	(8.6)	237	(22.3)	**0.013** ^†^
Stroke, *n* (%)	5	(8.6)	44	(4.1)	0.105^†^
Heart disease, *n* (%)	5	(8.6)	100	(9.4)	0.836^†^
Diabetes mellitus, *n* (%)	7	(12.1)	110	(10.3)	0.649^†^
Cancer, *n* (%)	2	(3.4)	85	(8.0)	0.210^†^
Depression (GDS score ≥6), *n* (%)	12	(20.7)	186	(17.5)	0.338
Cognitive function (MMSE score < 24), *n* (%)	13	(22.4)	154	(14.5)	0.058^†^
Body mass index					0.075^†^
<20.0, *n* (%)	11	(19.0)	158	(14.9)	
20.0–24.9, *n* (%)	27	(46.6)	652	(61.3)	
≥25.0, *n* (%)	20	(34.5)	253	(23.8)	
Blood nutritional parameters					
Albumin (<4.0), *n* (%)	8	(13.8)	132	(12.4)	0.796^†^
Hemoglobin					**0.022** ^†^
Male (<13.0), *n* (%)	4	(12.1)	39	(8.5)	
Female (<12.0), *n* (%)	6	(24.0)	49	(8.1)	
Total cholesterol					0.192^†^
Male (<160), *n* (%)	7	(21.2)	56	(12.2)	
Female (<180), *n* (%)	5	(20.0)	95	(15.7)	
Gait speed (m/s)					0.366^†^
<1.0, *n* (%)	8	(13.8)	118	(11.1)	
Grip strength					0.807^†^
Male (<26.0), *n* (%)	2	(6.1)	29	(6.3)	
Female (<18.0), *n* (%)	7	(28.0)	136	(22.5)	
Self‐perceived chewing ability					**<0.001** ^†^
Difficulties, *n* (%)	9	(17.3)	34	(3.2)	
Some difficulties, *n* (%)	31	(53.4)	310	(29.2)	
No difficulties, *n* (%)	16	(27.6)	702	(66.0)	

*Note*: Statistically significant *P*‐values are indicated using bold numbers. GDS, geriatric depression scale (15 items); MMSE, mini‐mental state examination (a 30‐point questionnaire); *n*, number of individuals; SD, standard deviation.

^†^ Chi‐squared test.

^‡^ *t*‐test.

### Kaplan–Meier estimate of LOI

**Figure 2:**
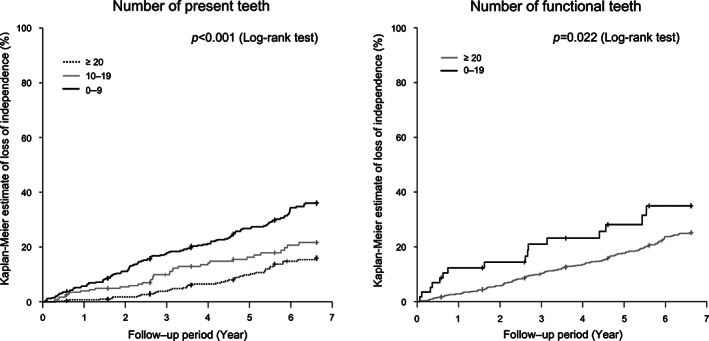
Kaplan–Meier estimates of the loss of independence categorized by numbers of present teeth (left) and functional teeth (right). Significant statistical differences were observed using log‐rank tests (number of present teeth: *P* < 0.001; number of functional teeth: *P* = 0.022).

During the follow‐up period, 158 participants received LTCI certification, and 47 participants died before receiving LTCI certification. Thus, the endpoint of this study was reached in 205 individuals during the follow‐up period. Figure 2 shows the Kaplan–Meier estimates of LOI among the study participants. Kaplan–Meier estimates of LOI in participants with smaller NPT and NFT were significantly greater than those in participants with larger NPT and NFT (log‐rank test, NPT: *P* < 0.001 and NFT: *P* = 0.022).

### Risk factors for LOI

Based on the results of the Kaplan–Meier analysis, further assessments were performed to test whether the NPT and NFT were independent risk factors for LOI, using Cox proportional hazard analysis (Table 2). In Model 0, smaller NPT (*P* < 0.001) and NFT (*P* = 0.036) were significant risk factors for LOI. However, a smaller NPT was not a significant risk factor for LOI in subsequent statistical models. In Model 1, a smaller NFT (*P* = 0.011) and older age (*P* < 0.001) were significant risk factors for LOI. In Model 2, a smaller NFT was not a significant risk factor, but older age (*P* < 0.001), male sex (*P* = 0.047), higher GDS score (*P* = 0.015), and lower MMSE score (*P* = 0.028) were significant risk factors for LOI. Detailed findings with respect to significant risk factors in subsequent statistical models were as follows.

In Model 3, as in Model 2, older age (*P* < 0.001), male sex (*P* = 0.042), higher GDS score (*P* = 0.023), and lower MMSE score (*P* = 0.019) were significant risk factors for LOI. In addition, a lower blood albumin level (*P* = 0.049) was identified as a significant risk factor.
In Model 4, older age (*P* < 0.001), male sex (*P* = 0.003), lower blood albumin (*P* = 0.030), and slower gait speed (*P* = 0.001) were significant risk factors for LOI.
In Model 5, lower self‐perceived chewing ability (*P* = 0.015) was identified as an additional significant risk factor for LOI. Other factors were older age (*P* < 0.001), male sex (*P* = 0.002), lower MMSE score (*P* = 0.014), lower blood albumin levels (*P* = 0.040), and slower gait speed (*P* = 0.006).

**Table 2:** Hazard ratios and 95% confidence intervals of each predictor for each Cox proportional hazard model

	Model 0	Model 1	Model 2	Model 3	Model 4	Model 5
	HR (95% CI)	HR (95% CI)	HR (95% CI)	HR (95% CI)	HR (95% CI)	HR (95% CI)
Present teeth						
≥20	1.0 [Reference]	1.0 [Reference]	1.0 [Reference]	1.0 [Reference]	1.0 [Reference]	1.0 [Reference]
10–19	1.484 (0.949–2.321)	1.134 (0.718–1.792)	1.173 (0.734–1.876)	1.219 (0.751–1.977)	1.352 (0.815–2.243)	1.397 (0.834–2.340)
0–9	**2.468** **(1.740–3.500)**	1.338 (0.921–1.943)	1.265 (0.862–1.857)	1.357 (0.920–2.002)	1.391 (0.918–2.109)	1.336 (0.868–2.055)
Functional teeth						
≥20	1.0 [Reference]	1.0 [Reference]	1.0 [Reference]	1.0 [Reference]	1.0 [Reference]	1.0 [Reference]
0–19	**1.739** **(1.036–2.921)**	**1.975** **(1.168–3.340)**	1.797 (0.995–3.245)	1.496 (0.801–2.795)	1.539 (0.805–2.941)	1.234 (0.617–2.468)
Age	–	**1.129** **(1.105–1.153)**	**1.129** **(1.104–1.155)**	**1.123** **(1.097–1.150)**	**1.111** **(1.084–1.140)**	**1.116** **(1.088–1.145)**
Sex						
Female	–	1.0 [Reference]	1.0 [Reference]	1.0 [Reference]	1.0 [Reference]	1.0 [Reference]
Male	–	1.254 (0.949–1.658)	**1.365** **(1.004–1.854)**	**1.378** **(1.011–1.877)**	**1.682** **(1.188–2.379)**	**1.758** **(1.234–2.505)**
History of chronic disease						
Hypertension	–	–	1.147 (0.845–1.556)	1.119 (0.816–1.536)	1.559 (0.829–1.622)	1.188 (0.844–1.673)
Hyperlipidemia	–	–	1.047 (0.701–1.562)	1.087 (0.722–1.636)	1.189 (0.776–1.820)	1.167 (0.759–1.795)
Stroke	–	–	1.794 (0.959–3.356)	1.609 (0.829–3.124)	1.463 (0.727–2.941)	1.243 (0.606–2.547)
Heart disease	–	–	1.176 (0.745–1.856)	1.140 (0.718–1.808)	1.089 (0.665–1.784)	1.156 (0.703–1.900)
Diabetes mellitus	–	–	1.453 (0.944–2.237)	1.369 (0.882–2.126)	1.564 (0.993–2.463)	1.556 (0.978–2.476)
Cancer	–	–	0.785 (0.456–1.352)	0.821 (0.471–1.432)	1.045 (0.601–1.816)	1.000 (0.559–1.788)
Depression						
GDS score < 6	–	–	1.0 [Reference]	1.0 [Reference]	1.0 [Reference]	1.0 [Reference]
GDS score ≥ 6	–	–	**1.512** **(1.084–2.108)**	**1.484** **(1.057–2.085)**	1.377 (0.952–1.993)	1.337 (0.914–1.955)
Cognitive function						
MMSE score ≥ 24	–	–	1.0 [Reference]	1.0 [Reference]	1.0 [Reference]	1.0 [Reference]
MMSE score < 24	–	–	**1.573** **(1.052–2.354)**	**1.634** **(1.084–2.464)**	1.549 (1.000–2.398)	**1.731** **(1.116–2.687)**
Body mass index						
≥25.0	–	–	–	1.0 [Reference]	1.0 [Reference]	1.0 [Reference]
20.0–24.9	–	–	–	0.926 (0.646–1.327)	1.031 (0.706–1.504)	1.120 (0.755–1.662)
<20.0	–	–	–	0.882 (0.548–1.421)	1.023 (0.623–1.679)	1.048 (0.634–1.732)
Blood nutritional parameters						
Albumin						
≥4.0	–	–	–	1.0 [Reference]	1.0 [Reference]	1.0 [Reference]
<4.0	–	–	–	**1.419** **(1.002–2.010)**	**1.496** **(1.040–2.152)**	**1.471** **(1.017–2.127)**
Hemoglobin						
Male (≥13.0), Female (≥12.0)	–	–	–	1.0 [Reference]	1.0 [Reference]	1.0 [Reference]
Male (<13.0), Female (<12.0)	–	–	–	1.233 (0.813–1.870)	1.071 (0.688–1.669)	1.084 (0.695–1.692)
Total cholesterol						
Male (≥160), Female (≥180)	–	–	–	1.0 [Reference]	1.0 [Reference]	1.0 [Reference]
Male (<160), Female (<180)	–	–	–	1.402 (0.984–1.998)	1.301 (0.900–1.881)	1.431 (0.984–2.083)
Gait speed (m/s)						
<1.0 m/s	–	–	–	–	**1.952** **(1.325–2.876)**	**1.750** **(1.170–2.617)**
Grip strength						
Male (≥26.0 kgf), Female (≥18.0 kgf)	–	–	–	–	1.0 [Reference]	1.0 [Reference]
Male (<26.0 kgf), Female (<18.0 kgf)	–	–	–	–	1.412 (0.983–2.029)	1.341 (0.925–1.946)
Self‐perceived chewing ability						
No difficulties	–	–	–	–	–	1.0 [Reference]
Some difficulties	–	–	–	–	–	0.778 (0.532–1.136)
Difficulties	–	–	–	–	–	**2.056** **(1.153–3.666)**

*Note*: Statistically significant (*P* < 0.05) HRs and 95% CIs are indicated using bold numbers.

CI, confidence interval; GDS, geriatric depression scale; HR, hazard ratio; MMSE, mini‐mental state examination.

HRs and 95% confidence intervals of NPT and NFT submitted as predictors for each Cox proportional model are shown in Table 2.

## Discussion

The NPT was previously reported as a significant risk factor for LOI.^5^ The present study investigated the relationship between the incidence of LOI and the NPT and NFT. Because individuals owning a prosthesis use it during meals regardless of their NPT, we predicted that the NFT reflects an individual's masticatory function more accurately than does the NPT. Therefore, the NFT and NPT were submitted simultaneously as predictors, and the models were adjusted for other variables.

Kaplan–Meier analysis revealed that concurrent smaller NPT and NFT significantly increased the estimated incidence of LOI. A smaller NPT at baseline increased the incidence of LOI, which was consistent with the findings of a previous study.^5^ A novel and extremely valuable finding of the present study is that lower NFT at baseline increased the incidence of LOI. Although NPT has been reported to predict mortality in previous systematic reviews,^22^, ^23^ NFT is known to be a stronger predictor of all‐cause mortality than NPT.^6^ Thus, the relationship between life expectancy, LOI, NPT, and NFT is consistent.

Subsequent Cox proportional hazard analyses demonstrated that older age, male sex, depression, lower cognitive and physical functions, and the presence of malnutrition were significant risk factors for LOI. These results are consistent with those of previous studies.^24^ This suggests that the findings obtained from this population exhibit high external validity. The results also suggest that a smaller NFT has a greater effect on the future incidence of LOI than a smaller NPT. Thus, the NFT may be a more accurate indicator of intraoral status and function, and the maintenance of the NFT may prevent future LOI. By adding depressive mood and cognitive function to Model 1, a statistical significance, which was observed in the NFT for LOI in Model 1, disappeared in Model 2. This finding indirectly demonstrates that depression and cognitive function may have been related to the NFT at baseline. Indeed, a recent study demonstrated that the loss of functional teeth and functional occlusal units was associated with increased cognitive impairment.^25^ Further studies are needed to clarify this interesting hypothesis.

Further, in Model 3, lower serum albumin level was a significant risk factor for LOI. Based on the results of the multivariate analysis, lower serum albumin level (<4.0) appears to be a stronger risk factor for LOI than the NFT. It can be assumed that a lower NFT could induce insufficient nutrition intake, leading to a higher incidence of LOI. However, baseline serum albumin levels did not differ between groups categorized by the NFT (Table 1). This indicates that serum albumin levels are not related to the NFT. It is well known that serum albumin levels are decreased in individuals with liver or renal dysfunction,^26^ and these conditions may also induce the incidence of LOI. Unfortunately, the functions of these organs were not evaluated in this study. Future analysis involving these parameters are needed to find an answer to this question.

In Model 5, lower self‐perceived chewing ability was a significant risk factor for LOI. Several studies have reported that lower self‐perceived chewing ability is significantly associated with the incidence of functional disability and the development of frailty.^1^, ^11^ In addition, a recent prospective study demonstrated that subjectively evaluated poor oral function, including chewing ability, is associated with a higher risk of LOI in functionally independent community‐dwelling older adults.^27^ The significant association between lower self‐perceived chewing ability and incidence of LOI found in this study is consistent with the previously reported findings. Thus, a decline in self‐perceived chewing ability can lead to the development of functional disability and frailty, thereby contributing to LOI. A previous systematic review reported a positive correlation between the number of teeth, functional tooth units, and subjective masticatory ability in older adults.^28^ Again, by adding self‐perceived chewing ability into the predictors of Model 5, the HR of NFT (0–19) decreased from 1.539 in Model 4 to 1.234 in Model 5, while the HR of NPT (0–9) showed almost no change. These statistical findings indirectly indicate that the NFT and self‐perceived chewing ability at baseline may have been correlated. In addition, it would be reasonable to expect that a lower NFT had a negative effect on the self‐perceived chewing ability at baseline, which could be a significant risk factor for the incidence of LOI. Furthermore, slower gait speed was an independent risk factor even in Model 5, supporting the notion that gait speed and subjective masticatory ability are independent risk factors for the incidence of LOI.^2^, ^3^, ^4^

This study has limitations. First, the statistical model did not include periodontal condition. Future analysis should include periodontal condition as one of the baseline predictors, because chronic inflammation could affect the NPT and the NFT as well as LOI.^29^ Second, denture quality was not assessed at baseline. Denture quality is known to affect an individual's nutritional status directly;^30^ therefore, future studies need to assess its impact on LOI. Third, it is important to note that the self‐perceived chewing ability observed in this study could be a measure for overall masticatory function, which would also affect the NPT, NFT, and denture quality. To specifically achieve the aim of the present study, which is to compare the relative risk of PT and FT on LOI, this study analyzed the relationships in four models (Model 0 to Model 4) without self‐perceived chewing ability or denture quality assessment. Nevertheless, self‐perceived chewing ability would be an informative variable with which to examine the effect of masticatory function on LOI. Finally, NPT and NFT were simultaneously submitted in all Cox proportional hazard models in this study. This may have caused over‐adjustment. Thus, we also submitted the two variables separately in the same analytical models (Model 1 to 5), and the results are presented in supplemental tables. The results show that a smaller NPT was not a significant risk factor for LOI in any model (Table S2). In contrast, a smaller NFT was a significant risk factor for LOI in Models 1 and 2 (Table S3). This suggests the smaller NFT was over‐adjusted. However, these findings strengthened the idea that the NFT at baseline is more closely related to future LOI. Furthermore, these results suggest that NPT and NFT were not affected by multicollinearity with each other.

In conclusion, this study found that LOI was more closely related to the NFT than to the NPT, which is a known risk factor. Moreover, the association between LOI and self‐perceived chewing ability was stronger than that between LOI and the NFT or NPT in community‐dwelling Japanese older adults. These results suggest that maintaining healthy masticatory function by keeping a high enough NFT is important for protection against the future incidence of LOI and preserving healthy nutrition.

## Disclosure statement

The authors declare no conflicts of interest.

## Author contributions

Study concept and design: KM, SS, TI. Acquisition of subjects and/or data: HH, KM, MR, KT, HY, TK. Preparation of the manuscript: KM, TI Editing the manuscript: SS, HY, TK.

## Supporting information


**Table S1.** Comparisons of baseline properties between the groups categorized by the future incidence of loss of independence.

**Table S2.** Hazard ratios and 95% confidence intervals of present teeth and each predictor for Cox proportional hazard models.

**Table S3.** Hazard ratios and 95% confidence intervals of functional teeth and each predictor for Cox proportional hazard models.

## Data Availability

The data that support the findings of this study are available from the corresponding author upon reasonable request.
